# Differential distribution shifts in two subregions of East Asian subtropical evergreen broadleaved forests—a case of Magnoliaceae

**DOI:** 10.3389/fpls.2023.1326207

**Published:** 2024-01-23

**Authors:** Hai-Yang Wu, Yue-Han Liu, Qiu-Xiang He, Jun-Wei Ye, Bin Tian

**Affiliations:** ^1^ Key Laboratory for Forest Resources Conservation and Utilization in the Southwest Mountains of China, Ministry of Education, Southwest Forestry University, Kunming, China; ^2^ National Plateau Wetlands Research Center, Southwest Forestry University, Kunming, China; ^3^ Yunnan Key Laboratory of Plateau Wetland Conservation Restoration and Ecological Services, Southwest Forestry University, Kunming, China

**Keywords:** 2070s, last glacial maximum, MaxEnt, mountains, refugia

## Abstract

**Aim:**

East Asian subtropical evergreen broad-leaved forests (EBLFs) are composed of western and eastern subregions with different topographical and environmental conditions. The distribution shifts over time of plants in the two subregions are predicted to be different, but the difference has seldom been investigated.

**Methods:**

Potential distributions of 53 Magnoliaceae species (22 in the western and 31 in the eastern subregion) during the last glacial maximum (LGM), present, and the 2070s were predicted using MaxEnt based on 58 environmental variables. The changes in the distribution range size and centroid over time were analyzed. Species-level potential habitats were overlaid to uncover species diversity distribution, and the distributions over time were overlaid to discover long-term refugia.

**Results:**

At present, the potential distributions are significantly larger than those shown by the occurrence points. During the LGM, 20/22 species in the western subregion experienced increases in range size through downwards and southward migrations, while decreases in range size in the eastern subregion (27/31 species) were accompanied by northward and eastward migrations. In the future, range size declines and northward shifts will both be found; northwestward shifts will exist in most (20/22 species) species in the western subregion, while both northwest- and northeastward shifts will occur in the eastern subregion. The diversity hotspots experienced a slight southward shift in the past and upwards to the mountain region in the future in the western subregion; in the eastern subregion, shrinks occurred in eastern China in the past and shrinks were shown in all regions in the future. Long-term refugia-preserving diversity was found in the mountains across the entire EBLFs region.

**Main conclusions:**

Significant differences in distribution shifts from past to present and similar distribution shifts from present to future are revealed in the two subregions. Species diversity in both subregions experienced no significant shifts from past to future, and Magnoliaceae plants could be preserved in mountainous regions throughout the EBLFs.

## 1 Introduction

**Figure 1:**
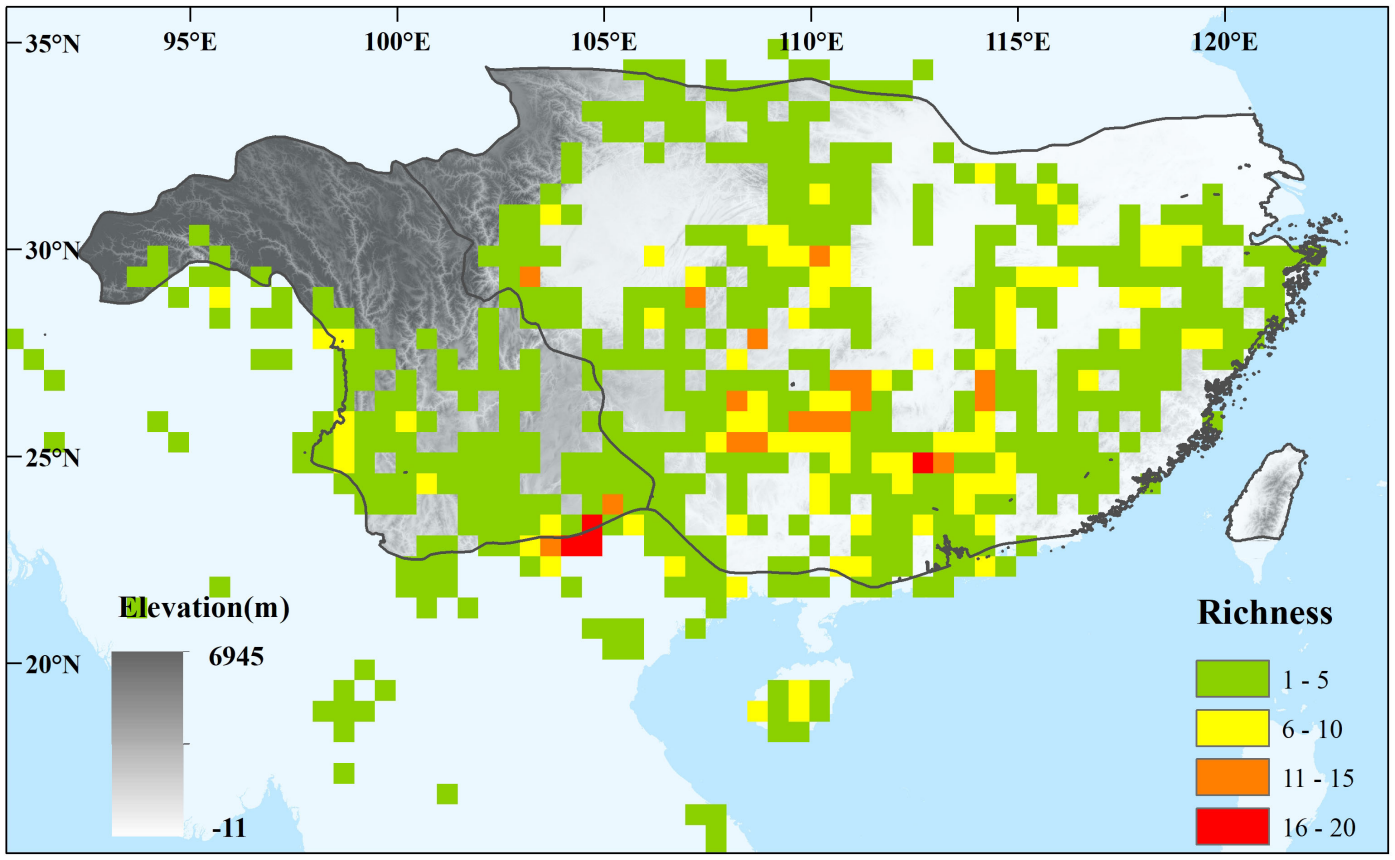
Species diversity of sampled 53 Magnoliaceae species inferred from occurrence points at 0.5° × 0.5° scale. The East Asian subtropical evergreen broadleaved forests (EBLFs) and their two subregions are labeled based on the division of subregion of the eastern humid evergreen broad-leaved forest (IV A) and the subregion of the western semi-humid evergreen broad-leaved forest (IV B) of the vegetation map of the People’s Republic of China (1: 1,000,000) (Zhang, 2007).

In East Asia, subtropical evergreen broadleaved forests (EBLFs), the most characteristic vegetation, currently occur between ca. 24–32°N latitude and 93–123°E longitude (**Figure 1**) (Wu, 1980; Zhang, 2007; Song and Da, 2016). The EBLFs harbor the highest level of species diversity and endemism (Huang et al., 2016; Xu et al., 2016) and are a significant refugia for Tertiary relicts (Tang et al., 2018). They fundamentally contribute to both the biodiversity and ecosystem services of East Asia (Wu and Wu, 1996). The assembly of EBLFs is profoundly affected by environmental changes during the Pleistocene, such as alternations of glacial–interglacial ages (Harrison et al., 2001; Ni et al., 2010), and in the future, such as global climate warming (Liang et al., 2018). Changes in distribution from the past to the present will deeper our understanding of the mechanism of biodiversity formation, while changes from the present to the future will improve the conservation of biodiversity. Modeling and predicting the distribution shifts of EBLFs have become increasingly important and are limited.

The past distribution of EBLFs can be inferred through pollen-based paleovegetation reconstruction or ecological niche modeling (ENM). Reconstructions show that the EBLFs are expected to significantly retreat southward (ca. 15–24°N) during the last glacial maximum (LGM) (Harrison et al., 2001; Ni et al., 2010). In *Cinnamomum* (Lauraceae), a dominant genus in EBLFs, Zhou et al. (2021a) found its potential distributions changes little during the past through ENM. Other species-level ENMs indicate that only a few species confirm the southern retreat pattern during the LGM, whereas more species show potential distributions in the northern region above 24°N (Qiu et al., 2011; Ye et al., 2017b). In response to climate warming in the future, the overall distribution pattern of species diversity or endemism pattern of Theaceae will not change significantly in 2070s (Zhang et al., 2020), while at species level, less species would experience range expansions and more species experience range contractions (during 2050s and 2070s) (Tang and Zhao, 2022), causing some species become threatened (Zhang et al., 2020). However, Zhang (2007) divided EBLFs (IV) into two subregions (IV A and IV B) based on regional differences caused by different environmental conditions and floral components (**Figure 1**), while, possible different distribution changes through time of plants in the two subregions have seldom been explored, as previous studies have considered EBLFs as a whole.

In the two subregions, eastern EBLFs components (dominated by Sino-Japanese elements, rich in relict and endemic species) are distributed at low latitudes of 100 m–1,500 (2,800 m), while western EBLFs components (mainly Sino-Himalayan elements, rich in endemic species) are distributed in high mountains at 1,500 m–2,500 m (2,800 m) with highly heterogeneous topography and environment (Song and Da, 2016). The eastern subregion is mainly influenced by the East Asian monsoon and the western subregion by the Indian monsoon, and climatic differences exist in both temperature or precipitation (Chen et al., 2015; Song and Da, 2016). Thus, the distribution shifts over time are likely to differ. During the LGM, plants were predicted to migrate to lower latitudes or altitudes to trace suitable climates (Hewitt, 2004). Hypothetically, western evergreen species can survive cooler and drier glacial ages by altitudinal downward shift, which could lead to the expansion of range size as a result of a monotonic increase in surface area when moving down mountainsides (La Sorte and Jetz, 2010), but this may not be the case in the eastern subregion (Qiu et al., 2011; Liu et al., 2012). When facing future climate warming, reduced range sizes are expected to shift to higher altitudes or latitudes (Parmesan, 2006; Zhang et al., 2020). However, range size increases are possible in the west subregion because of the complex topography of the Hengduan Mountains (HMS) or the escape shelter role played by the Qinghai–Tibet Plateau (QTP) during upward and westward shifts (Liang et al., 2018). Distinct genetic lineages revealed by the phylogeographic of biogeographic studies further indicate different topographical and environmental conditions in the two subregions (Meng et al., 2017; Ye et al., 2017a). Therefore, whether distribution shifts in the two subregions occur differently requires further investigation.

Magnoliaceae was selected for the case study. First, many Magnoliaceae plants in the EBLFs are mainly distributed only in the eastern or western subregions (http://www.iplant.cn/). Song and Da (2016) considered it be a dominant family in EBLFs, except Fagaceae, Lauraceae, Theaceae, and Hamamelidaceae, leading to its representativeness in studying the biodiversity of EBLFs (Hai et al., 2022). Finally, among the approximately 120 Magnoliaceae species in China (Xia et al., 2008), approximately 70% are threatened according to the China Biodiversity Red List (Xie et al., 2022), their potential distributions in the future are significant for the conservation of biodiversity. Therefore, potential distributions of Magnoliaceae plants were predicted using MaxEnt (Phillips et al., 2006) in three different time periods (LGM, present, and the 2070s) to infer (1) whether species in the eastern and western subregions respond differently to environmental changes, (2) distribution pattern of species diversity and area of species richness hotspots at different time periods, and (3) long-term refugia preserving the diversity of Magnoliaceae species.

## 2 Materials and methods

### 2.1 Research area and species occurrence data

We selected the region within approximately 15°N–35°N and 90° E–125° E as the research area. First, the current EBLFs occur between 24°N and 32°N latitudes and 93°E–123°E longitudes (**Figure 1**). Vegetation reconstructions indicate a significant southward retraction to 15°N–24°N during the LGM and a northward shift (200 km–500 km) in eastern China during the mid-Holocene (Harrison et al., 2001; Ni et al., 2010). In the future, the potential distribution of Theaceae (Zhang et al., 2020) and *Cinnamomum* (Zhou et al., 2021a) will show no significant northward shift of the northern boundary of the EBLFs. Therefore, the region shown in **Figure 1** was selected as the research area to cover the potential distribution shift of Magnoliaceae species from the past to the future.

Native Chinese Magnoliaceae species distributed mainly in the EBLFs region were selected. Species occurrence points with accurate geographical coordinates were obtained from the National Specimen Information Infrastructure (NSII, http://www.nsii.org.cn/) and Global Biodiversity Information Facility (GBIF, http://www.gbif.org/). For species with limited data, the data were supplemented by referring to relevant literature. Magnoliaceae species distributed in both the eastern and western subregions were excluded, while occurrence points outside the EBLFs were also included to precisely predict the potential distributions (Phillips et al., 2006). As species are more likely to be recorded in areas with easy access and high interest, while less recorded in hard-to-reach and low-interest areas, spatial filters can randomly remove localities that are within given distance from one another, thus improving the performance of ENM (Boria et al., 2014). Spatial filtering was performed using the R package spThin (Aiello-Lammens et al., 2015) at a scale of 1 km × 1 km.

### 2.2 Environmental variables

The environmental conditions to which plants respond include climatic, topographic, and edaphic factors (Huang et al., 2021). In addition, UV-B radiation has an important effect on the distribution of some plants (Li et al., 2019; Wang et al., 2019). Hence, we compiled 58 environmental variables, including elevation, 19 climatic factors, 32 soil variables, and six UV-B variables (**Supplementary Table 1**). The 19 bioclimatic variables were downloaded from the WorldClim dataset (http://www.worldclim.org/) with a resolution of 30 s (2.5 min in LGM). During the LGM, the Community Climate System Model 4 (CCSM4) (Shields et al., 2012) model was selected because it is one of the most efficient models for predicting the influence of climatic change (Abdelaal et al., 2019) and has been widely used in previous studies on EBLFs (Wang et al., 2015; Ye et al., 2019). The future climate under the most typical concentration trajectory, RCP8.5, which represents a highly concentrated one, was selected. The 32 soil variables were obtained from the Harmonized World Soil Database (30 s) (HWSD, http://www.fao.org/soils-portal/data-hub/en/), and UV-B variables were obtained from glUV (http://www.ufz.de/gluv/) (15 min). All data were resampled to a spatial resolution of 30 s. The same elevation, soil variables, and UV-B variables during the LGM and 2070s were used as the present (Li et al., 2019). Given that correlations and collinearity between environmental variables can lead to overfitting of the model, we chose the correct combination of variables for each species (see below for details).

### 2.3 Niche modeling

The maximum entropy modeling technique, MaxEnt 3.4.13 (Phillips et al., 2006) was used to predict the potential distributions. Several steps are performed to filter the variables. First, all 58 variables at present and the default parameters were used for the simulation. Then, variables with model contribution percentages of less than 0.2 were removed. For the two variables whose correlation is greater than 0.75, or Variance Inflation Factor (VIF) is greater than 5, only the variable that exerted more contribution was retained. Finally, the remaining variables were used to model the niche of each species. The three variables that showed the highest contributions were integrated to infer the most important variables.

In MaxEnt, we optimized the model using the ‘ENMevaluate’ function in the R package ENMeval (Muscarella et al., 2015). To enhance the transferability of the model, only the linear (L) and quadratic (Q) feature functions were selected (Elith et al., 2010), and 0.2, 0.4, 0.6, 0.8, 1, 1.5, 2, 2.5, 3, 3.5, 4, 4.5, and 5 were evaluated for the Regularization Multiplier (RM). The contribution of each environmental variable was evaluated using jackknife testing and a set cross-validation function with logistic outputs. For species with ≥25 occurrence records, cross-validation was applied. When occurrence records consisted of 5 or <25 records, we applied jackknife testing (Tang et al., 2018).

The receiver operating characteristic curve (ROC) area under the curve (AUC) was used to evaluate performance (Fawcett, 2006) according to different model repetition methods. For models constructed using the cross-validation method, the continuous Boyce index (CBI) was used to evaluate the performance. For models constructed using the folding knife method, leave-one-out was used to evaluate the performance (Chloe et al., 2015). The folding knife method evaluates the model performance based on the success rate (q, the percentage of correct predictions) and statistical significance.

The maximum training sensitivity plus specificity strategy (MSS) was used to demarcate the habitat (presence) and non-habitat (absence) of the species (Liu et al., 2016). Next, the arithmetic results from MaxEnt were loaded into ArcGIS 10.2 to perform a fitness classification and visualization expression, which enabled us to generate the potential distributions of Magnoliaceae plants.

### 2.4 Shifts of distribution and diversity over time

To obtain a species richness distrirbution map, we overlaid the presence/absence maps of different species, where the accumulated value for each grid represents the number of species. The queried coordinate points were overlaid to obtain a map of the observed species richness. Based on the queried coordinate points, the grid size was set to 0.5° x 0.5°.

We overlaid the habitat of all species from past to future to obtain refugia, defined as environmentally suitable areas that allowed the persistence of plants from past glacial–interglacial alternations and future global warming (Tang et al., 2018) in both the western and eastern subregions. In addition, we analyzed shifts of the distribution centroids of each species across different periods. All the analyses mentioned above were conducted using the SDMtools (Brown et al., 2017) plugin of ArcGIS. Potential range size changes from LGM to present (LGM-present), and present to future (2070s–present) were calculated as (α − β)/β, as ‘α’ represents range size of species during the LGM or 2070s and ‘β’ represents range size of species at present.

## 3 Results

### 3.1 Model performance and variable contributions

We collected 4,304 valid distribution points with accurate geographical coordinates for the 76 Magnoliaceae species that were mainly distributed in the study region (**Supplementary Table 2**). After excluding species with few distribution points (N <5, n = 11) and poor model performance (CBI/q <0.75, n = 7), 58 models were obtained. From these models, 31 (3,232 points) and 22 (777 points) species were selected from the eastern and western subregions, respectively, as the remaining five species were distributed in both subregions (**Supplementary Table 3**; **Supplementary Figures 1**, **2**). Thirty (56.6%) species are recorded as threatened species in the Chinese red list (http://www.iplant.cn/rep/protlist/4, Access date: 10 December 2023) including three Critically endangered (CR, three in the western subregion), eight Endangered (EN, five and three in eastern and western subregion, respectively) and 19 Vulnerable (VU, nine and 10 in eastern and western subregion, respectively) (**Supplementary Table 4**).

The average CBI/q value for the 53 species models was 0.95, with a minimum value of 0.75, indicating that the potential distributions could be properly predicted (**Supplementary Table 3**). The most important variables was the Mean Diurnal Range (bio2) and Min Temperature of Coldest Month (bio6) in the eastern and western subregions, respectively. Soil type (SU_CODE90) was the second most significant variable in both subregions (**Supplementary Figure 3**).

### 3.2 Distribution shifts in western and eastern subregions

**Figure 2:**
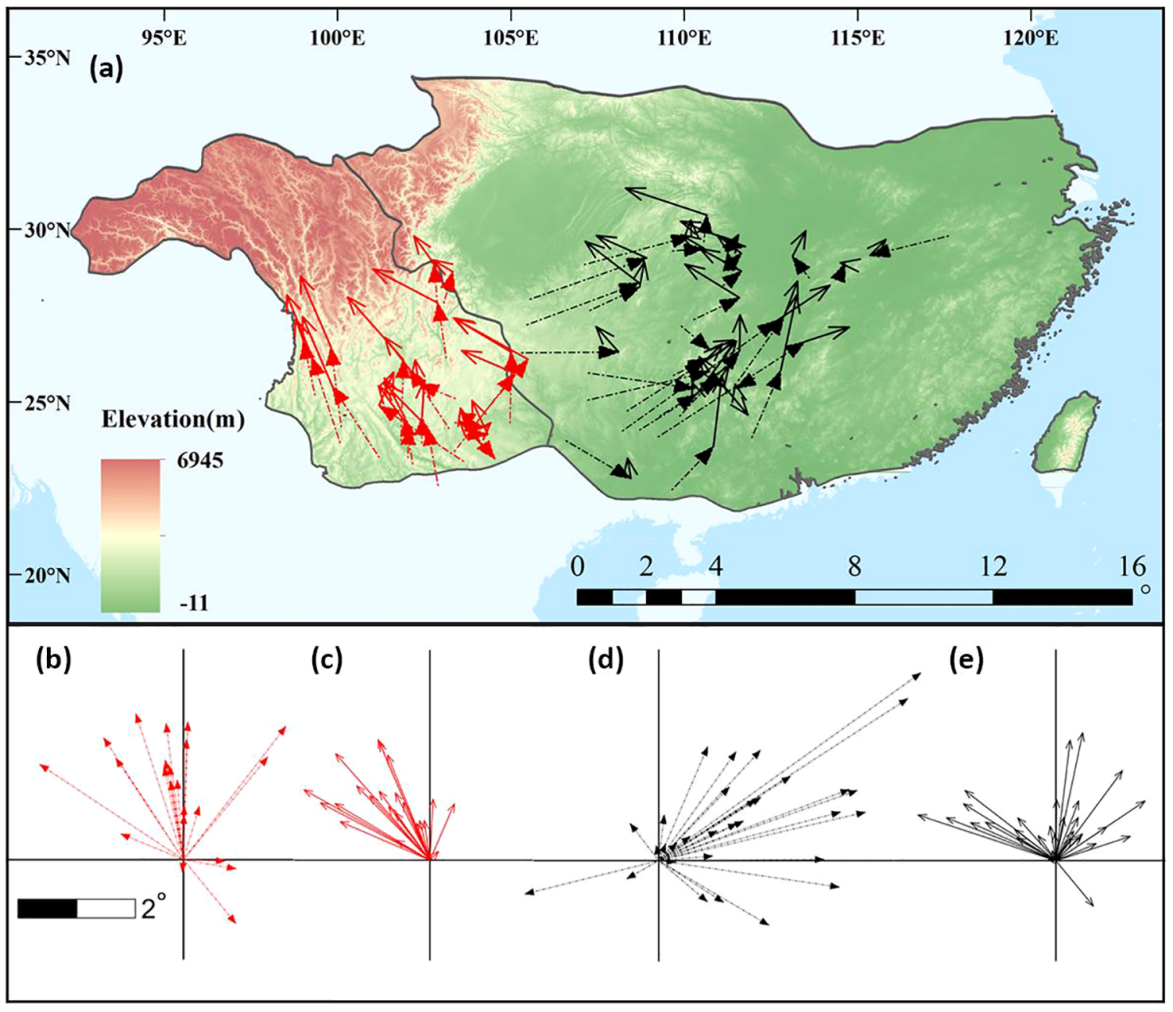
A comparison of centroid movements during different periods in the eastern and western subregions. **(A)** Distance and direction of change at the centroid of climatic niche. The red and black arrows represent the western and eastern subregions, respectively, and the dashed lines and solid lines represent movements from the last glacial maximum (LGM) to present and from present to 2070s, respectively. **(B–E)** summarize the distance and direction of shift for the studied plants with western **(B, C)** and eastern **(D, E)** subregions from the LGM to the present **(B, D)** and from the present to the 2070s **(C, E)**.

**Figure 2** shows the geographical changes in potential distribution centroids. From the LGM to the present, most (18/22, 81.8%) species in the western subregion experienced northward shifts, whereas most (28/31, 90.3%) eastern species experienced an eastward shift (**Figures 2A, B, D**; **Supplementary Table 4**). From the present to the 2070s, all western and almost all (30/31, 96.8%) eastern species underwent northward shifts (**Figures 2A, C, E**), and northwestward shifts were found in most species (20/22) in the western subregion, while both northwest- and northeastward migrations were found in the other subregion (**Supplementary Table 4**). **Supplementary Figure 4** shows the elevation, latitude, and longitude changes of the potential distribution centroids. The elevation of the distribution centroids changes little in the eastern subregion, while upslope trends are shown in the western subregion from the past to the future (**Supplementary Figures 4A, D**). Latitudinal changes show northward shifts since the LGM to the 2070s in both subregions (**Supplementary Figures 4B, E**). Westward longitudinal changes occurred continuously in the western subregion, whereas an eastward shift from the LGM to the present and no changes from the present to the 2070s were found in the eastern subregion (**Supplementary Figures 4C, F**).

**Figure 3:**
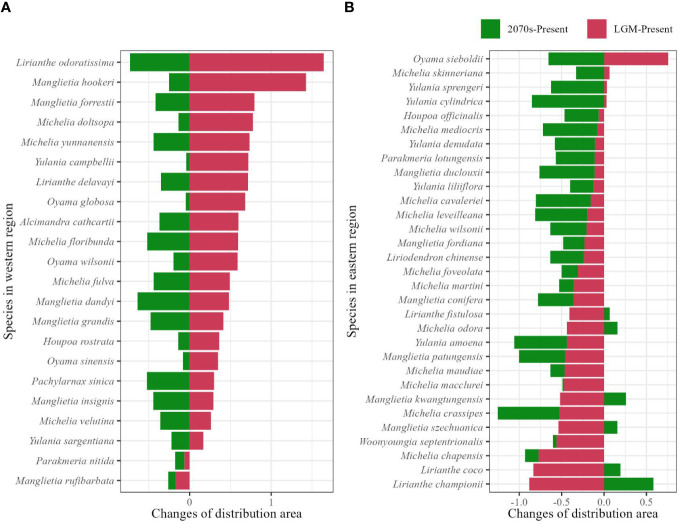
Range size changes of the potential distributions from the last glacial maximum to the present (LGM-present, red color), and from the present to the future at the 2070s (2070s–present, green color) in the western **(A)** and eastern **(B)** evergreen broadleaved forest subregions calculated by (α − β)/α, where 'α' represents range size of species at the LGM or the 2070s, and 'β' represents the range size of species at present.

From the past (LGM) to the present, shrinkage of the distribution area was found in 20/22 (90.9%) species, range from 0.17 to 1.64 times compared to the distribution area at present in the western subregion (**Figure 3A**; **Supplementary Table 4**), and expansions occurred in 27/31 (87.1%) species with a range of 0.06 to 0.88 times in the eastern subregion (**Figure 3B**; **Supplementary Table 4**). In the future, decrease of distribution area are both shown in western (22/22, 100%) and eastern (25/31, 80.6%) subregions, range from 0.04 to 0.73 and 0.02 to 0.85 times, respectively, and the remaining six species in eastern subregion undergo range size increase (**Figures 3A, B**; **Supplementary Table 4**).

Species were classified into different International Union for Conservation of Nature (IUCN) Red List categories based on habitat loss: Extinct (EX), CR, EN, VU, and Low Risk (LR) represented 100%, >80%, >50%, >30%, and <30% habitat loss, respectively (IUCN, 2001). Of the 30 threatened species, 24 (80%) continuously lost their habitat, and 14 of the remaining 23 unthreatened species became threatened as they experienced habitat loss of over 30%.

### 3.3 Species diversity changes in the two subregions

**Figure 4:**
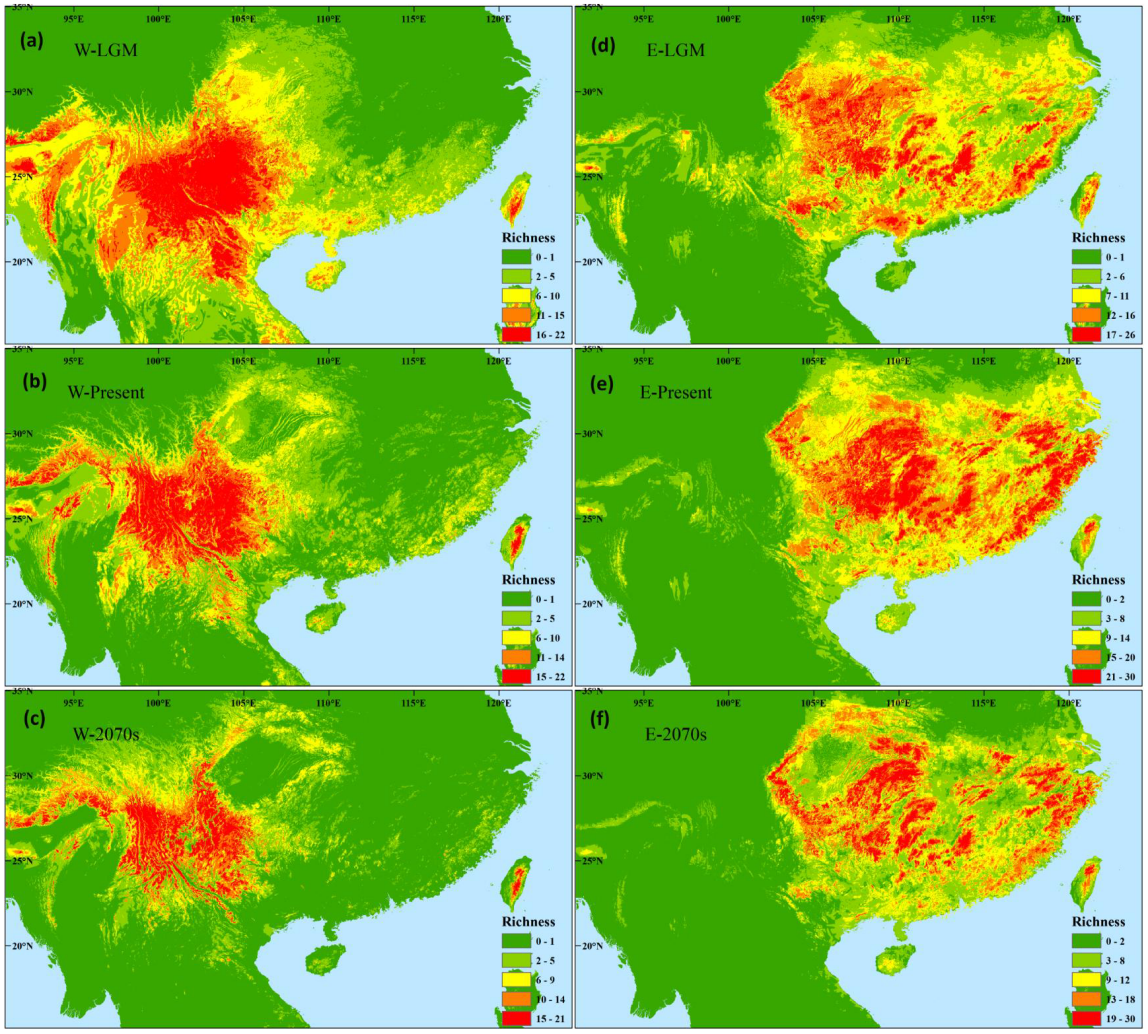
Differences in the potential species richness distribution of Magnoliaceae plants in the western **(A–C)** and eastern **(D–F)** evergreen broadleaved forest subregions at the last glacial maximum **(A, D)**, during the present **(B, E)**, and in the future of the 2070s **(C, F)** under the scenarios of RCP 8.5.

In the western subregion, species occurrence data showed high species diversity in the western HMS and southeastern margin (**Figure 1**; **Supplementary Figures 1**, **2**). The ENMs indicated high species diversity across the entire subregion, and higher diversity was found in the mountain region compared to the valley region (**Figure 4A**). During the LGM, similar and slightly southward shifts in species hotspots were found, and many species occupied the valley region (**Figure 4B**). A similar species distribution pattern was found in the future, whereas more severe contractions occurred in the mountain region (**Figure 4C**).

In the eastern subregion, higher species diversity was found in the mountains in central, southern, and eastern China through species occurrence data and ENMs (**Figures 1**, **4E**; **Supplementary Figures 1**, 2). More diversity hotspots were found in the western than in the eastern area during the LGM (**Figure 4D**). In the future, similar diversity distribution pattern and significant shrinks will be observed (**Figure 4F**).

**Figure 5:**
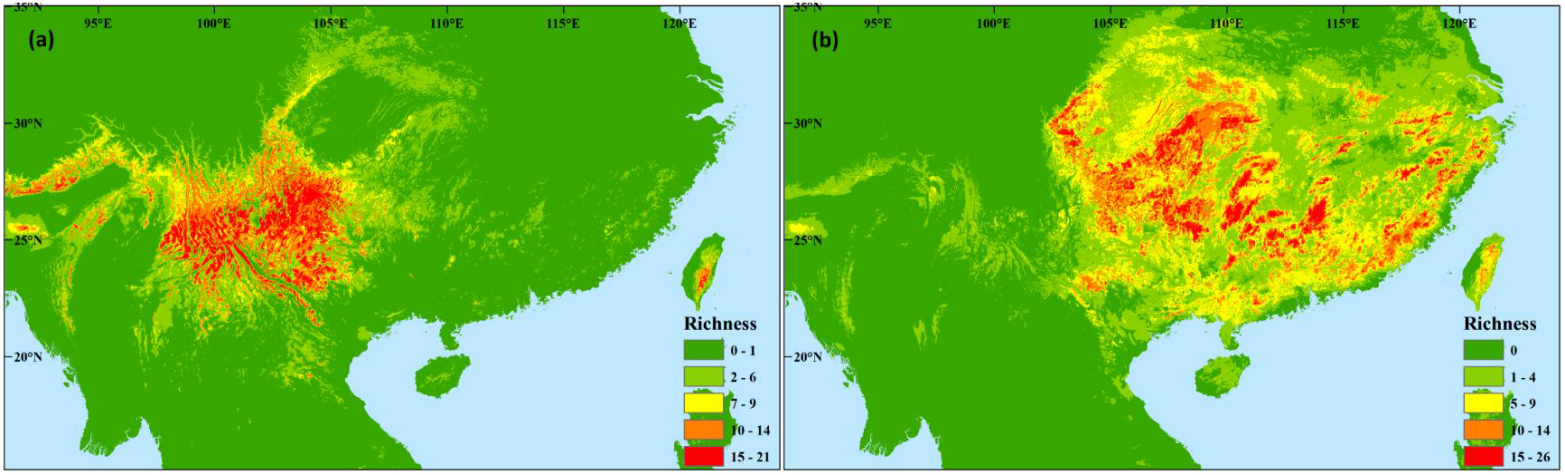
Overlap of potential richness of Magnoliaceae species in the three different periods (last glacial maximum, present, and the 2070s) of the **(A)** western subregion and **(B)** eastern subregion.

The refugia of western Magnoliaceae plants are distributed across the HMS and western Yunnan-Guizhou Plateau (YGP) (**Figure 5A**). Eastern Magnoliaceae plants can survive long-term in central (eastern YGP and Xuefeng Mountain), southern (Nanling Mountain), and eastern (Dabie-Tianmu Mountain and Wuyi Mountain) China (**Figure 5B**).

## 4 Discussion

### 4.1 Differential distribution shifts between western and eastern subregions

According to the predicted potential distributions of Magnoliaceae species at three different periods, we found that plants in the western and eastern subregions experienced different shift and range size change patterns from past to present, while similar trends occur from present to future; differences also exist in environmental variables that show significant contributions.

From the LGM to the present, Magnoliaceae species experience decrease in range sizes in the western subregion while increase in the eastern subregion. Western species show downwards (to lower altitudes) and southward retreat to longitudinal range-gorge regions during the glacial ages (Liu et al., 2012). More surface at lower altitudes (La Sorte and Jetz, 2010) or even connections of isolated high-latitude populations during interglacial ages in valley regions resulted in increases of range sizes during the LGM (**Figure 4A**). In contrast, in the HMS, north of the western EBLFs subregion, Liang et al. (2018) found larger range sizes at present compared to the LGM, which may be explained by the complex topography of the HMS or the escape shelter role played by the QTP during upward and westward shifts. In the eastern subregion, species tend to migrate northward and eastward from the LGM to the present, whereas changes in elevation are not significant, accompanied by an increase in range size. These increases may be due to range expansions from glacial refugia in the YGP or mountains in Central China (Qiu et al., 2011; Liu et al., 2012). Expansion can also be found in other dominant EBLFs species, such as *Loropetalum chinense* (Gong et al., 2016), *Lindera aggregata* (Ye et al., 2019), and *Machilus thunbergii* (Fan et al., 2016).

In the future, range size declines and northward shifts will be found in both subregions; northwestward shifts will exist in most species in the western subregion, while both northwest- and northeastward shifts will occur in the eastern subregion. Migration to higher latitude is common to trace suitable habitats under future climate warming (Parmesan, 2006). Less survival possibility north of 32°N would result in range size shrinks of EBLFs species. Further support has been received from plants of *Magnolia* (Wang et al., 2022), Theaceae species (Zhang et al., 2020; Tang and Zhao, 2022) or some case studies, such as *Magnolia sprengeri* (Zhou et al., 2021b) and *Glyptostrobus pensilis* (Pueyo-Herrera et al., 2022). Similar to *Millettia*, a few Magnoliaceae species experience range size expansions that may be caused by increased precipitation (Zhao et al., 2023). In the western subregion, the pattern of northeastward escape of western Magnoliaceae species to higher elevations is similar to that of HMS plants, and the possibility of size expansion exists if the escape reaches the QTP (Liang et al., 2018). However, both westward and eastward migration occurred in the eastern subregion. Although the elevation of habitat changes little, mountains in central and eastern China may provide a more suitable microenvironment for Magnoliaceae species (Qiu et al., 2011; Liu et al., 2012), and the Nanling Mountains in south China could act as a dispersal corridor (Tian et al., 2018).

Unlike other dominant families, such as Lauraceae (Liao et al., 2022), Fagaceae (Hai et al., 2022) and Theaceae (Tang and Zhao, 2022), whose distributions are affected by both temperature and precipitation variables, Magnoliaceae plants are influenced mainly by temperature and soil variables. The most contributing variable also varied in the western (bio6) and eastern (bio2) subregions. Another important finding is that the soil contributes significantly in both subregions. Higher model performance and more accurate potential distribution prediction can be achieved by adding soil variables (Ray et al., 2016), but only a limited number of studies have included soil variables in EBLFs (Cui et al., 2016). Differences in soil types not only exist at different latitudes but also at different altitudes (Chen et al., 2015), and soil variables should be added in future studies to predict the potential distributions of species in EBLFs.

### 4.2 Plant diversity changes of Magnoliaceae from past to future

The distribution of species diversity was inferred through overlaid species-level potential distributions from past to future and it was found that the species diversity pattern changed little over time in both the western and eastern subregions, and long-term climate-stable refugia preserving Magnoliaceae plants were found in mountains across the entire EBLFs region.

At present, the species diversity pattern derived from occurrence data is similar to that of Xie et al. (2022), who compiled distribution data of 114 Magnoliaceae species, with higher diversity in the southeastern part of the western subregion and mountains in central and southern China of the eastern subregion. According to niche modeling, regions with higher diversity are widely found in both western and eastern subregions, such as eastern HMS, eastern YGP, and east China. A much lower observed diversity may be caused by unevenness in specimen collection (Xu et al., 2021), limited dispersal ability (Gherghel et al., 2020) and so on. During the LGM, extensions to low latitude in western subregion occur and extensive shrinks only happen in eastern China in eastern subregion, which are in contrast to reconstructed palaeovegetations which recover southwards retreat to ca. 24°N (Yu et al., 2000; Ni et al., 2010). In *Cinnamomum* (Lauraceae), another dominant taxon, ENM also showed no significant shifts during the LGM (Zhou et al., 2021a). Further support comes from case phylogeographic studies; more species can survive in refugia at northern of 24°N, and few species confirm a southward retreat (Ye et al., 2017b). This indicates that the EBLFs may not have experienced significant southward shifts during the LGM. In the future, shrinks of high diverse areas to mountain regions will be found in both regions, similar to other taxa in subtropical China, such as *Cinnamomum* (Zhou et al., 2021a) and Theaceae (Zhang et al., 2020; Tang and Zhao, 2022), as shifts to higher latitudes and elevations are general in response to future climate change (Parmesan, 2006; Liang et al., 2018).

Refugia analysis indicated that mountains across the entire EBLFs region can provide long-term stable environment for the survival of Magnoliaceae species. Similar to the present study, Tang et al. (2018) revealed that Southwest China (including Sichuan Basin, HMS, and YGP) and South China are long-term (LGM to 2070s) climatically stable refugia preserving relict species. In HMS plants, diversity is preserved long-term in HMS, although the refugia area is much more constricted than that in the present study (Liang et al., 2018). The regions of south and southwest China below 24°N also have higher species diversity of *Cinnamomum* at different time periods (Zhou et al., 2021a). Phylogeographic studies have shown that mountains in eastern China can serve as glacial refugia (Qiu et al., 2011; Liu et al., 2012). The centers of plant endemism identified through the distribution patterns of 555 endemic taxa further indicate higher endemism levels in mountains (López-Pujol et al., 2011). Relief terrain with environmental gradients and multiple vegetation belts in mountain systems can provide long-term stable environment for the survival of Magnoliaceae species or other EBLFs’ components (Muellner-Riehl, 2019).

Magnoliaceae species in the EBLFs will become more threatened in the future, and a proper conservation strategy is needed. Fourteen species with habitat loss over 30% are proposed to be added to the Red List in the future, resulting in 44/53 (83%) threatened species. The EBLFs have the most conservation gaps for protecting Magnoliaceae diversity hotspots (Xie et al., 2022) and may become worse in the 2070s. Mountains in southwest, central, and southern China are priority areas for protection not only for *in situ* but also for *ex situ* conservation (**Figure 5**). The Magnoliaceae community in eastern China is more vulnerable to environmental changes, and much attention is needed here as the populations may harbor high levels of genetic diversity (Fan et al., 2018).

## 5 Conclusions

Differences in distribution shifts in different periods between the two subregions of the EBLFs were investigated using ENMs of Magnoliaceae species based on accurate occurrence points and abundant environmental variables. Shift patterns and range size changes differ from past to present, while similar shift patterns are revealed from present to future. No significant changes in species diversity were found in different time periods in either subregions or mountains across the entire EBLFs, which can serve as long-term refugia preserving Magnoliaceae diversity. More Magnoliaceae species are expected to be threatened in the future, and proper conservation strategies are needed. The distribution shifts of EBLFs are complicated and more studies investigating potential distribution changes are needed for dominant or other taxa.

## Data availability statement

The original contributions presented in the study are included in the article/**Supplementary Material**. Further inquiries can be directed to the corresponding author.

## Author contributions

H-YW: Writing – original draft, Writing – review & editing. Y-HL: Writing – original draft, Writing – review & editing. Q-XH: Writing – original draft, Writing – review & editing. J-WY: Writing – original draft, Writing – review & editing. BT: Writing – original draft, Writing – review & editing.

## Supplementary material

Supplementary Figure 1 Species diversity of all 76 Magnoliaceae species inferred from occurrence points at 0.5 × 0.5° scale. The East Asian subtropical evergreen broadleaved forests (EBLFs) and its two subregions are labeled based on division of subregion of eastern humid evergreen broad-leaved forest (IV A) and subregion of western semi-humid evergreen broad-leaved forest (IV B) of Vegetation map of the People’s Republic of China (1: 1 000 000) (Zhang, 2007).

Supplementary Figure 2 Species diversity of sampled 22 and 31 Magnoliaceae species in western **(A)** and eastern **(B)** subregions, respectively, inferred from occurrence points at 0.5 × 0.5° scale. The East Asian subtropical evergreen broadleaved forests (EBLFs) and its two subregions are labeled by black solid line.

Supplementary Figure 3 The variables that contributes the most to Magnoliaceae in western **(A)** and eastern **(B)** subregion. V1(red), v2(blue) and v3(green) respectively represent the top three variables that contribute the most to each model. The different variables are shown in **Supplementary S3**.

Supplementary Figure 4 Changes in the distribution centroids of the climatic niche of 22 western **(A–C)** and 31 eastern **(D–F)** Magnoliaceae species from the last glacial maximum to 2070s (RCP8.5). **(A, D)** Trend in change in mean elevation. **(B, E)** Trend in change in mean latitude. **(C, F)** Trend in change in mean longitude.
