# Supplementary figures and images for: Differential distribution shifts in two subregions of East Asian subtropical evergreen broadleaved forests—a case of Magnoliaceae
Source: Front Plant Sci. 2024 Jan 23;14:1326207. doi: 10.3389/fpls.2023.1326207 (PMC10844446; doi:10.3389/fpls.2023.1326207)

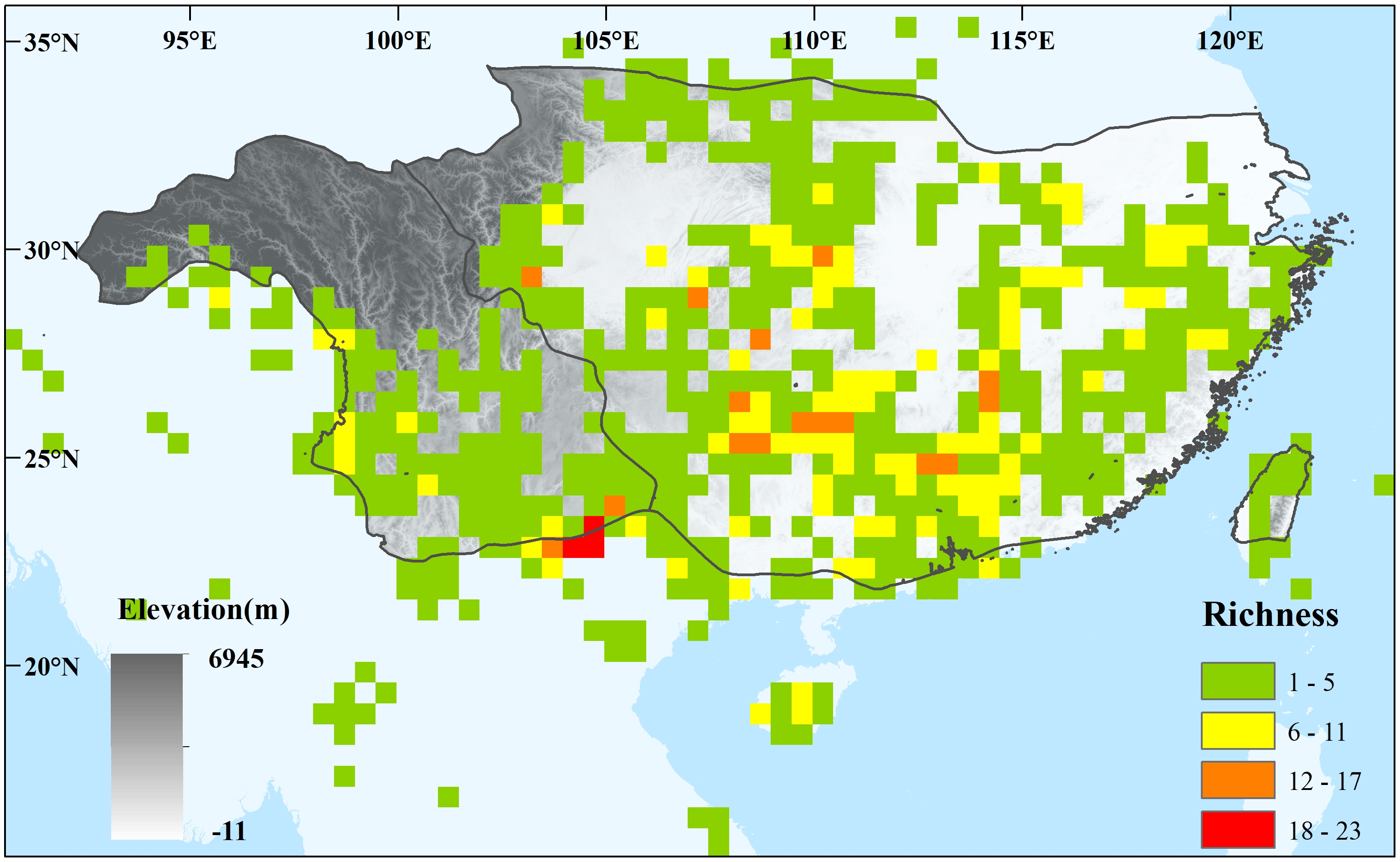

Supplement: Supplementary Figure 1 — Species diversity of all 76 Magnoliaceae species inferred from occurrence points at 0.5 × 0.5° scale. The East Asian subtropical evergreen broadleaved forests (EBLFs) and its two subregions are labeled based on division of subregion of eastern humid evergreen broad-leaved forest (IV A) and subregion of western semi-humid evergreen broad-leaved forest (IV B) of Vegetation map of the People’s Republic of China (1: 1 000 000) (Zhang, 2007). [file Image_1.jpeg]

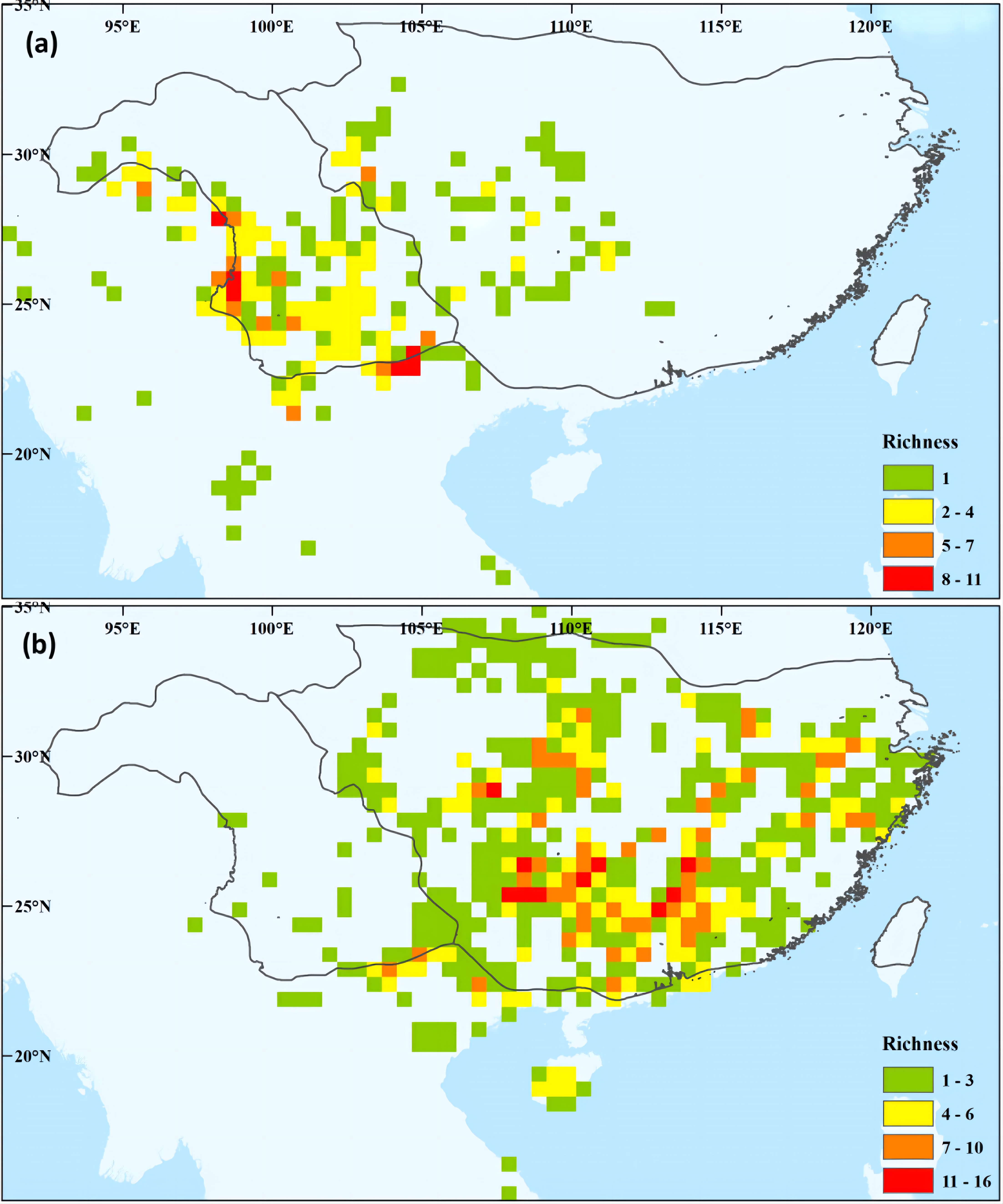

Supplement: Supplementary Figure 2 — Species diversity of sampled 22 and 31 Magnoliaceae species in western (A) and eastern (B) subregions, respectively, inferred from occurrence points at 0.5 × 0.5° scale. The East Asian subtropical evergreen broadleaved forests (EBLFs) and its two subregions are labeled by black solid line. [file Image_2.jpeg]

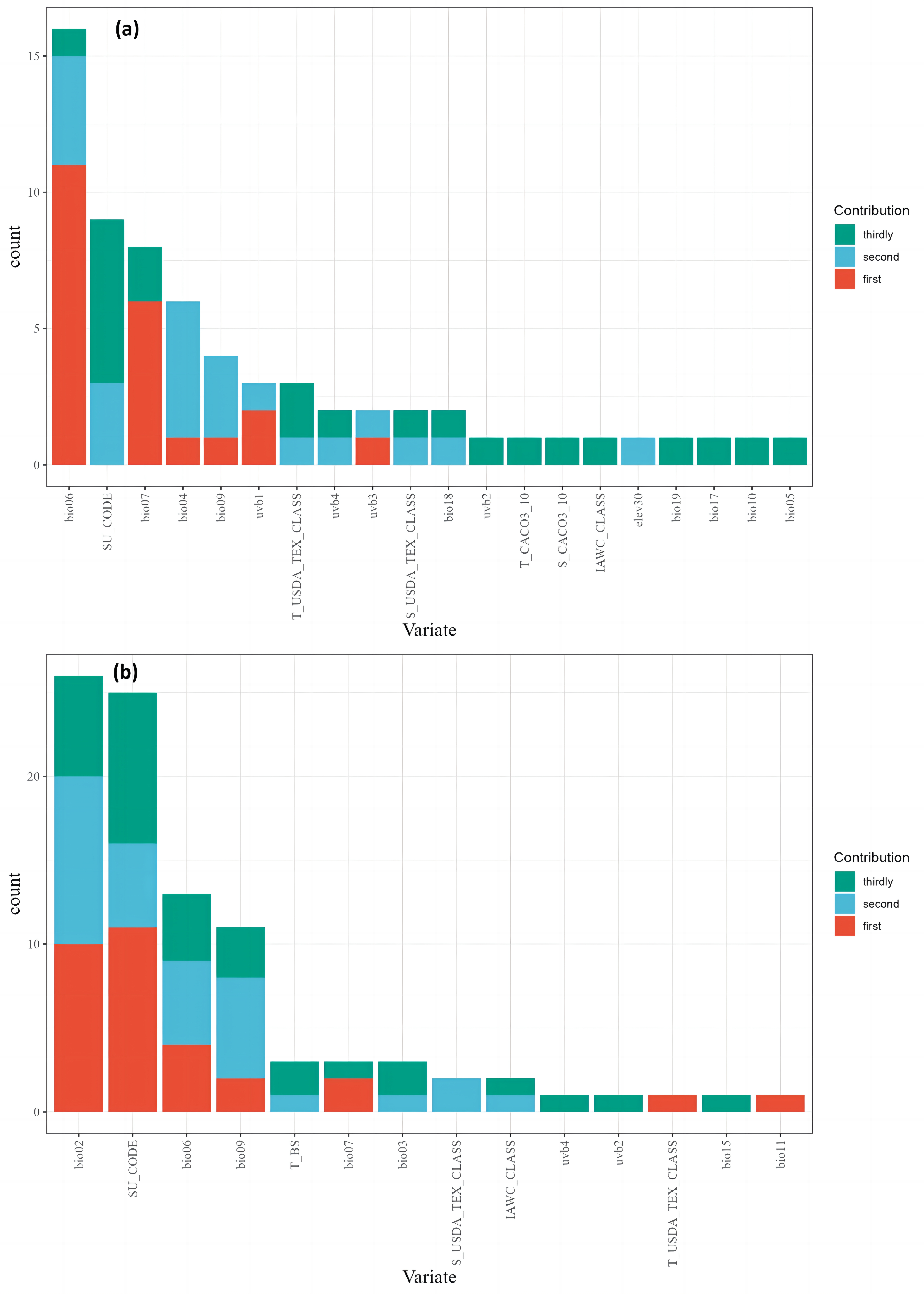

Supplement: Supplementary Figure 3 — The variables that contributes the most to Magnoliaceae in western (A) and eastern (B) subregion. V1(red), v2(blue) and v3(green) respectively represent the top three variables that contribute the most to each model. The different variables are shown in Supplementary S3. [file Image_3.jpeg]

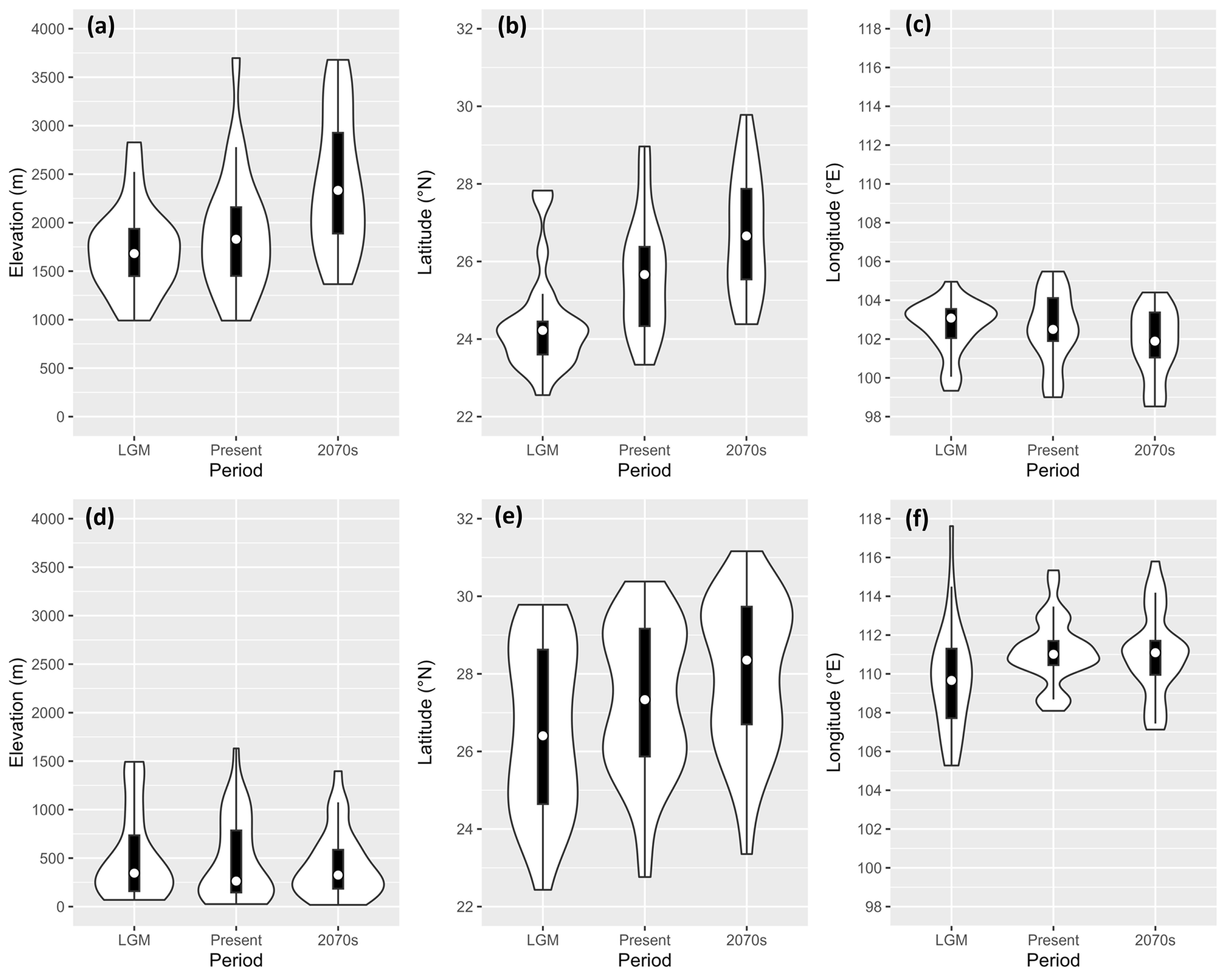

Supplement: Supplementary Figure 4 — Changes in the distribution centroids of the climatic niche of 22 western (A–C) and 31 eastern (D–F) Magnoliaceae species from the last glacial maximum to 2070s (RCP8.5). (A, D) Trend in change in mean elevation. (B, E) Trend in change in mean latitude. (C, F) Trend in change in mean longitude. [file Image_4.jpeg]
